# Human polyomavirus BKPyV and JCPyV serostatus has no impact on women´s human papillomavirus infection outcome

**DOI:** 10.3389/fcimb.2023.1190019

**Published:** 2023-06-02

**Authors:** Hanna K. Laine, Tim Waterboer, Kari Syrjänen, Seija Grenman, Karolina Louvanto, Stina Syrjänen

**Affiliations:** ^1^ Department of Oral and Maxillofacial Diseases, University of Helsinki, Helsinki, Finland; ^2^ Department of Oral Pathology and Radiology, Faculty of Medicine, University of Turku, Turku, Finland; ^3^ Division of Infections and Cancer Epidemiology, German Cancer Research Center (DKFZ), Heidelberg, Germany; ^4^ SMW Consultants Ltd, Kaarina, Finland; ^5^ Department of Obstetrics and Gynecology, University of Turku, Turku University Hospital, Turku, Finland; ^6^ Department of Obstetrics and Gynecology, Faculty of Medicine and Health Technology, Tampere University, Tampere, Finland; ^7^ Department of Obstetrics and Gynecology, Tampere University Hospital, Tampere, Finland; ^8^ Department of Pathology, University of Turku, Turku University Hospital, Turku, Finland

**Keywords:** polyomavirus, papillomavirus, seroprevalence, cytology, cervix, mouth, outcome, longitudinal study

## Abstract

**Introduction:**

Polyomaviruses have both structural and functional similarities with papillomaviruses. Accordingly, their role in human papillomavirus (HPV) associated malignancies has been studied with conflicting results. Our goal was to disclose any association between BK (BKPyV) and/or JC (JCPyV) polyomavirus serology and HPV data derived from Finnish women (327) in a 6-year prospective follow-up.

**Methods:**

Glutathione S-transferase fusion-protein-capture (ELISA) in combination with fluorescent bead technology was used to analyze antibodies to BKPyV and JCPyV. In the longitudinal setting, BKPyV or JCPyV serostatus was related to i) oral- and ii) genital low (LR)- and high risk (HR) HPV DNA detection, iii) HPV16 persistence at both these sites, iv) results of the Pap (Papanicolaou) smear taken at baseline, and v) development of incident CIN (cervical intraepithelial neoplasia) during the follow-up.

**Results:**

Being BKPyV or JCPyV seropositive was not significantly associated with HPV seropositivity to either LR- or HR-genotypes, genital- or oral HPV DNA positivity, persistence of genital- or oral HPV16 infection, grade of Pap smear, or development of incident CIN.

**Discussion:**

Thus, the present study could not provide any confirmation to the concept that co-infections by HPyV and HPV have interactions that impact on the clinical manifestations or outcomes of HPV infections either in the genital tract or in the oral mucosa.

## 1 Introduction

Human polyomaviruses (HPyV) and human papillomaviruses (HPV) are closely related viruses with remarkable structural and functional similarities. HPyVs are non-enveloped DNA viruses with circular double-stranded genome varying from 5100 to 5400 base pairs (Walker et al., 2019). Currently HPyV family comprises 13 members of which the best known are BK polyomavirus (BKPyV) (Gardner et al., 1971) and JC polyomavirus (JCPyV) (Padgett et al., 1971). Polyomavirus carriage can persist for lifetime, mostly as subclinical infections.

Also HPVs are small, non-enveloped double-stranded DNA viruses of which currently more than 200 genotypes have been identified. Based on their different risk to induce precancer lesions and cancers, HPV genotypes are further categorized as low- (LR) or high-risk (HR) HPV genotypes (Vats et al., 2021). Both HPyVs and HR-HPVs are oncogenic viruses provided with oncoproteins, large T-antigen and E6 and E7, respectively. These viruses do interfere with the functions of p53 and pRb (Guidry and Scott, 2017; Vats et al., 2021).

It has been suggested that during their simultaneous infection (either at same location or different), polyomaviruses could potentiate the transforming properties of HPVs (Guidry and Scott, 2017). However, recent *in vitro* studies on the mutual interactions between polyoma- and papillomaviruses are scanty. However, some clues on the possible interaction between these two viruses are provided by i) the older *in vitro* studies on SV-40 performed with HPV-positive cell lines, e.g. HeLa (HPV18-positive) and Caski (HPV16-positive) or ii) Cos-1 cell line, the latter containing one copy of SV40. Of particular interest is the study by Wildeman and coworkers (1989) on large T-antigen, which is required to start viral replication (Wildeman, 1989). The authors showed that in HeLa cells, large T-antigen trans-activated the SV40 early promoter as well as the late promoter on replication-defective templates. Trans-activation was also seen, when a large T-antigen, unable to be transported to the nucleus was used, and it was still able to transform cells. This simultaneous trans-activation of early and late promoters was not found in mouse embryonal carcinoma cells (Wildeman, 1989).

Until now, the simultaneous presence of both BKPyV or JCPyV and HPV has been analyzed in genital, colorectal, head and neck, or lung lesions (Giuliani et al., 2007; Giuliani et al., 2008; Comar, 2011; Fraase et al., 2012; Alosaimi et al., 2014; Polz-Gruszka et al., 2015; Poluschkin et al., 2018). The results of these studies have been conflicting, and no firm conclusions can be drawn on the suggested co-operation between polyoma- and papillomaviruses in human.

The Finnish Family HPV Study has been ongoing since 1998 in Turku University, Finland, focusing on HPV dynamics related to oral and genital infection and immunology in a longitudinal study setting within regular Finnish families (Rintala et al., 2005; Rintala et al., 2006; Syrjänen et al., 2009). In the original setting, 327 women (mean age 25.5 ± 3.4 years), pregnant at their baseline visit, and 131 of their spouses (mean age 28.8 ± 5.0 years), as well as 331 newborns were enrolled in the cohort and followed up for 72 months. A detailed medical history and sexual practices were recorded, in addition to extensive serial sampling samples (sera, saliva, placenta, breast milk, and semen). This cohort offers an ideal setting to study the impact of HPyV on HPV infection and its outcome, if any (Rintala et al., 2005; Rintala et al., 2006; Syrjänen et al., 2009).

In the present study, we analyzed the association between BKPyV and/or JCPyV serostatus (positive/negative) and several key HPV-related covariates in women participants from the longitudinal Finnish Family HPV Study. These HPV-covariates of interest include HPV serology and DNA status for both LR- and HR-HPV types, persistence of HPV16 infection in oral- and genital mucosa, Pap smear abnormalities, and incident CIN during the six-year follow-up.

## 2 Materials and methods

### 2.1 Participants

In total, 327 of the 329 women enrolled originally to the Finnish Family HPV Study, were eligible in this study. The participants were pregnant in their third trimester when enrolled at the Maternity Unit of the Turku University Hospital, Turku, Finland (Rintala et al., 2005). The participants filled in a standardized questionnaire providing information on the demographics as previously summarized (Rintala et al., 2005). Mean age of the participants was 25.5 years (range, 18-38 years). None of the participants have been vaccinated to HPV because the study cohort was recruited before the era of HPV vaccination. The study was approved by the Research Ethics Committee of Turku University Hospital (#3/1998, with amendments 45/1801/2018).

### 2.2 Serology

#### 2.2.1 Blood samples

Blood samples from these mothers were collected at baseline (n=327) and at 12(n=278)-, 24(n=220)-, and 36(n=261) months - visits as described earlier (Syrjänen et al., 2009). The samples were centrifuged at 1150 g for 10 min (Sorvall GLC-2; DuPont Instrument). Each serum sample was immediately divided into three 1 ml aliquots and stored first at -20°C for no longer than one week and then at -70°C until shipped on dry ice to DKFZ, Heidelberg, Germany, for serological analysis.

#### 2.2.2 Pap smear

A routine Pap (Papanicolaou) smear was taken at the baseline visit and at 12-month, 24-month, 36-month, and 6-year follow-up visits with the conventional 3-sample technique with wooden spatulas and a brush (Cytobrush, MedScand, Sweden) as described earlier (Louvanto et al., 2010; Louvanto et al., 2011). The slide was fixed with a preservative (Spray-Cyte; Becton Dickinson and Company, Sparks, Md.). For classification, the Bethesda System (TBS) was used.

#### 2.2.3 Brush samples from genital and oral mucosa for HPV testing

Scrapings for HPV DNA testing were taken from the cervical and oral mucosa by using a brush (Cytobrush®, MedScan, Malmö, Sweden) as described earlier (Louvanto et al., 2010; Louvanto et al., 2011; Rautava et al., 2012). Oral brush samples were placed in a tube containing 80% ethanol, while the media for the cervical brush samples was 0.05 M phosphate-buffered saline (PBS) supplemented with 100 μg of gentamicin. The samples were immediately frozen (−20°C) and then stored at −70°C. The cervical scrapings for HPV testing were obtained at baseline and at the 2-month, 12-month, 24-month, 36-month, and 6-year follow-up visits. Oral scrapings were taken at similar intervals with an additional sampling at 2-month visit.

#### 2.2.4 Analysis of BKPyV and JCPyV serology

The samples were analysed for antibodies to VP1 of BKPyV and JCPyV. The antibody-detection method was based on glutathione S-transferase fusion-protein (GST)-capture ELISA (Sehr et al., 2001) in combination with fluorescent bead technology (Waterboer et al., 2005). The method for BKPyV and JCPyV serology has been described in more detailed previously (Antonsson et al., 2010; Gossai et al., 2016). Sera were scored as positive when the antigen-specific MFI values were 400 MFI or for VP1 antigen of BKPyV and JCPyV. The selection of these cut-off values is described more detailed in reference 23 (Gossai et al., 2016). We also selected another, highly stringent cut-off levels of 5000 MFI for BKPyV and 2000 for JCPyV (Michael et al., 2008). Our speculation was that these high antibody levels could be sign of an ongoing viral replication of BKPyV or JCPyV. In the present study we used only the baseline BKPyV and JCPyV serology data of the pregnant women.

#### 2.2.5 Analysis of HPV serology

The samples were analyzed for antibodies to the major capsid protein L1 of LR-HPV types 6, 11 and HR-HPV types 16, 18, and 45 by multiplex HPV serology based also on glutathione S-transferase fusion-protein capture on fluorescent beads (Waterboer et al., 2005; Waterboer et al., 2006). Sera were scored as HPV-positive when the antigen-specific MFI values were greater than the cut-off level of 200 MFI for L1 antigen of individual HPV types as described earlier (Syrjänen et al., 2009). Similarly, only the first visit (baseline) HPV serology data was included in this analysis.

#### 2.2.6 HPV testing

HPV DNA was extracted from the oral and genital brush samples with the high salt method as described previously (Miller et al., 1988). HPV-testing for the presence of any HR-HPVs was performed using nested PCR using MY09/MY11 as external and GP05+/GP06+ as internal primers. HPV genotyping was performed with a Multimetrix kit^®^ (Multimetrix, Progen Biotechnik GmbH, Heidelberg, Germany) which detects 24 LR- and HR-HPV-genotypes as follows: LR-HPV6, 11, 42, 43, 44, and 70; and HR-HPV16, 18, 26, 31, 33, 35, 39, 45, 51, 52, 53, 56, 58, 59, 66, 68, 73, and 82 as described earlier (Schmitt et al., 2006; Louvanto et al., 2010; Rautava et al., 2012).

#### 2.2.7 HPV16 persistence

All HPV16 DNA positive samples (at any visit) were first identified as described earlier (Rautava et al., 2012; Louvanto et al., 2014). Among the HPV16-positive cases, persistence was recorded when HPV16 DNA was detectable at consequent follow-up samples for a minimum of 24 months (Louvanto et al., 2014). The HPV16 non-persistence category includes cases where HPV16 infection did not persist for 24 months, or cleared without any persistence during the follow-up.

#### 2.2.8 Incident cervical intraepithelial neoplasia

Following two cervical smears taken at 6–12 month intervals containing abnormal atypical squamous cells of undetermined significance or worse (ASCUS+), women were referred for colposcopy and biopsies to confirm the cytological findings as described earlier (Louvanto et al., 2011). All biopsies were fixed in formalin, embedded in paraffin and processed for hematoxylin and eosin-stained sections following routine procedures. The slides were re-evaluated to confirm the diagnosis of CIN (Louvanto et al., 2011).

### 2.3 Statistical analysis

All statistical analyses were run by using the SPSS for Windows (version 28.0.1.1; SPSS, Inc.) software package. Conventional 2x2 tables were used to analyze categorical variables, tested using the likelihood-ratio test or Fisher’s exact. Differences in the means of continuous variables were analyzed by ANOVA or Mann–Whitney/Kruskal–Wallis test for two and multiple independent samples, respectively. Univariate logistic regression was used to calculate odds ratio (OR) and 95% confidence intervals (CI) between two binary variables. All statistical tests performed were two-sided and declared to be significant when p<0.05.

## 3 Results

The median and mean IgG-antibody levels of BKPyV in serum of 327 pregnant women were 8640 MFI with a range from 72 to 20773 MFI and 8497 ± 416 MFI, respectively. The corresponding IgG-antibody levels of JCPyV were lower, median being 765 MFI with a range from 0 to 15007 MFI and mean ± SD 2340 ± 2901MFI. The mean IgG antibody levels of BKPyV were nearly four-fold higher than those of JCPyV. Also the standard deviation for BKPyV IgG antibodies is nearly seven times lower than that for JCPyV.

**Table 1** shows the associations between BKPyV/JCPyV serology at the baseline visit, stratified by HPV serology, HPV DNA positivity (both, baseline) and HPV16 persistence in genital and oral mucosa, Pap smear results, and incident CIN found during the six years follow-up. The HPV specific results of The Finnish Family HPV Study have been published earlier. Thus, the original reference with regard to the HPV data is given in the respected subtitle of the **Table 1**.

**Table 1:** BKPyV (MFI 400) and JCPyV (MFI 400) serostatus stratified by key HPV-related variables.

HPV-related variables	BKPyV +	BKPyV-	p-value	JCPyV+	JCPyV-	p-value
HPV Serostatus^ ref (Syrjänen et al., 2009)						
LR-HPV+	86(93.5%)	6(6.5%)	0.875	59(64.1%)	33(35.9%)	0.223
HR-HPV+	36(97.3%)	1(2.7%)		25(67.6%)	12(32.4%)	
LR+/HR+	86(95.6%)	4(4.4%)		53(58.9%)	37(41.1%)	
*HPV-	102(94.4%)	6(5.6%)		56(51.9%)	52(48.1%)	
Genital DNA status (baseline)^ ref (Louvanto et al., 2010)						
HR-HPV+	223(95.7%)	10(4.3%)		137(58.8%)	96(41.2%)	
LR-HPV+	20(90.9%)	2(9.1%)	0.338	10(45.5%)	12(54.5%)	0.280
**HPV-	49(94.2%)	3(5.8%)		34(65.4%)	18(34.6.)	
Oral DNA status (baseline)^ ref (Rautava et al., 2012)						
HR-HPV+	144(94.1%)	9(5.9%)		87(56.9%)	66(43.1%)	
LR-HPV+	17(100%)	0(0.0%)	0.846	11(64.7%)	6(35.3%)	0.727
**HPV-	149(94.9%)	8(5.1%)		95(60.5%)	62(39.5%)	
Genital HPV16 persistence ^ref (Louvanto et al., 2010)						
Yes	82(96.5%)	3(3.5%)	1.000	50(58.8%)	35(41.2%)	1.000
No	70(95.9%)	3(4.1%)		43(58.9%)	30(41.1%)	
Oral HPV16 persistence ^ref (Rautava et al., 2012)						
Yes	55(90.2%)	6(9.8%)	0.272	36(59.0%)	25(41.0%)	0.467
No	58(96.7%)	2(3.3%)		31(51.7%)	29(48.3%)	
Baseline Pap (TBS)^ref (Louvanto et al., 2011)						
Normal	275(94.8%)	15(5.2%)		171(59.0%)	119(41.0%)	
ASC-US	25(100%)	0(0.0%)	0.094	15(60.0%)	10(40.0%)	1.000
Low SIL	8(80.0%)	2(20.0%)		6(60.0%)	4(40.0%)	
Incident CIN^ ref (Louvanto et al., 2011)						
Yes	11(91.7%)	1(8.3%)	0.479	8(66.7%)	4(33.3%)	0.767
No	299(94.9%)	16(5.1%)		185(58.7%)	130(41.3%)	

BKPyV +, BK polyomavirus positive; BKPyV-, BK polyomavirus negative; JCPyV+, JC polyomavirus positive; JCPyV-, JC polyomavirus negative; HR-HPV+, high-risk human papillomavirus (HPV) positive; LR-HPV+, low-risk HPV positive; *HPV-, always seronegative to both LR- and HR-HPV; **HPV-, HPV DNA-negative; TBS, the Bethesda System; ASC-US, atypical squamous cells of undetermined significance; SIL, squamous intraepithelial lesion; CIN, cervical intraepithelial neoplasia.

The original HPV data is given as the reference to the respective subtitle (^ref).

### 3.1 BKPyV and JCPyV serology versus HPV serology

Of the HR-HPV seropositive women (IgG-antibodies to HPV16, 18, and/or 45 L1), 97.3% and 67.6% were also BKPyV- and JCPyV seropositive (MFI 400). The BKPyV and JCPyV seroprevalence (MFI 400) was practically identical among LR-HPV seropositive women (IgG-antibodies to HPV6 and/or HPV11 L1), 93.5% and 64.1%, respectively. Next we used the very stringent MFI values for BKPyV seropositivity (MFI 5000) and for JCPyV (MFI 2000) **Table 2**. This resulted in lower rates of seropositivity to both viruses, as expected, but no statistically significant association with HPV serology could be seen. However, p-value declined between BKPyV and HPV serology (from 0.875 to 0.442) while no changes were found between JCPyV and HPV serology (**Table 2**).

**Table 2:** BKPyV (MFI 5000) and JCPyV (MFI 2000) serostatus stratified by key HPV-related variables.

HPV-related variables	BKPyV +	BKPyV-	p-value	JCPyV+	JCPyV-	p-value
HPV Serostatus^ ref (Syrjänen et al., 2009)						
LR-HPV+	60(65.2%)	32(34.8%)	0.441	36(39.1%)	56(60.9%)	0.237
HR-HPV+	25(67.6%)	12(32.4%)		18(48.6%)	19(51.4%)	
LR+/HR+	25(64.4%)	32(35.6%)		34(37.8%)	56(62.2%)	
*HPV-	80(74.1%)	28(25.9%)		33(30.6%)	75(69.4%)	
Genital DNA status (baseline)^ ref (Louvanto et al., 2010)						
HR-HPV+	162(69.5%)	71(30.5%)		90(38.6%)	143(61.4%)	
LR-HPV+	14(63.6%)	8(36.4%)	0.819	7(31.8%)	15(68.2%)	0.391
**HPV-	37(71.2%)	15(28.8%)		15(28.8%)	37(71.2%)	
Oral DNA status (baseline)^ ref (Rautava et al., 2012)						
HR-HPV+	107(69.9%)	46(30.1%)		55(35.9%)	98(64.1%)	
LR-HPV+	13(76.5%)	4(23.5%)	0.553	6(35.3%)	11(64.7%)	0.912
**HPV-	103(65.6%)	54(34.4%)		60(38.2%)	97(61.8%)	
Genital HPV16 persistence ^ref (Louvanto et al., 2010)						
Yes	56(65.9%)	29(34.1%)	0.497	30(35.3%)	55(64.7%)	0.742
No	52(71.2%)	21(28.8%)		28(38.4%)	45(61.6%)	
Oral HPV16 persistence ^ref (Rautava et al., 2012)						
Yes	39(63.9%)	22(36.1%)	0.237	26(42.6%)	35(57.4%)	0.187
No	45(75.0%)	15(25.0%)		18(30.0%)	42(70.0%)	
Baseline Pap (TBS)^ref (Louvanto et al., 2011)						
Normal	200(69.0%)	90(31.0%)		107(36.9%)	183(63.1%)	
ASC-US	17(68.0%)	8(32.0%)	0.484	8(32.0%)	17(68.0%)	0.617
Low SIL	5(50.0%)	5(50.0%)		5(50.0%)	5(50.0%)	
Incident CIN^ ref (Louvanto et al., 2011)						
Yes	6(50.0%)	6(50.0%)	0.208	6(50.0%)	6(50.0%)	0.371
No	217(68.9%)	98(31.1%)		115(36.5%)	200(63.5%)	

BKPyV +, BK polyomavirus positive; BKPyV-, BK polyomavirus negative; JCPyV+, JC polyomavirus positive; JCPyV-, JC polyomavirus negative; HR-HPV+, high-risk human papillomavirus (HPV) positive; LR-HPV+, low-risk HPV positive; *HPV-, always seronegative to both LR- and HR-HPV; **HPV-, HPV DNA-negative; TBS, the Bethesda System; ASC-US, atypical squamous cells of undetermined significance; SIL, squamous intraepithelial lesion; CIN, cervical intraepithelial neoplasia.

The original HPV data is given as the reference to the respective subtitle (^ref).

### 3.2 BKPyV and JCPyV serology versus HPV DNA status

BKPyV and/or JCPyV serostatus was stratified by HR- and LR-HPV DNA status (at baseline) as well as HPV16 persistence in genital or oral mucosa (**Tables 1**, **2**). Nearly all BKPyV seropositive women carried HR- or LR-HPV DNA both in the genital (95.7% and 90.9%, respectively) and oral mucosa (94.1% and 100.0%, respectively) (**Table 1**). HR- or LR-HPV DNA positivity in the genital (58.8% and 45.5%, respectively) or oral mucosa (56.9% and 64.7%, respectively) was less prevalent among JCPyV seropositive women (**Table 1**).

No statistically significant differences in BKPyV seroprevalence were observed among women with and without persistent genital- or oral HPV16-infection, and the same was true with JCPyV serostatus as well. No statistically significant differences between BKPyV and JCPyV serology and HPV DNA data were found even the stringent cut-off for BKPyV and JCPyV seropositivity was used (**Table 2**).

### 3.3 BKPyV and JCPyV serology versus cervical cytology and CIN

Of the 327 women, 35 had cytological abnormalities (ASC-US, and SIL) in their Pap smear taken at the entry to the study when pregnant. ASC-US and low SIL was detected in 33 and 21 BKPyV- and JCPyV-seropositive women, respectively. However, as nearly all women with normal cytology were also seropositive to BKPyV (95%) and 59% were JCPyV seropositive, the differences did not reach statistical significance (p=0.094). During the follow-up, 12 women developed an incident CIN. Similarly as found with cytology most of these women were seropositive to BKPyV and 58.7% were JCPyV seropositive (**Table 1**). **Table 2** shows that when using the stringent cut-offs for BKPyV and JCPyV seropositivity the number of women with abnormal cytology declined from 33 to 22 in the BKPyV seropositive group and from 21 to 8 in JCPyV seropositive women. Neither the incident CIN could be associated with BKPyV and JCPyV seropositivity.

In assessing the risk estimates (OR) of being BKPyV or JCPyV seropositive, none of the HPV-related covariates proved to be significant predictors (**Table 3**). In the other way round, baseline BKPyV- and/or JCPyV seropositivity does not seem to be significantly associated with HPV DNA detection, HPV16 persistence, Pap smear abnormalities or progression to incident CIN during a long-term prospective follow-up.

**Table 3:** Association between BKPyV and JCPyV seropositivity with HPV serostatus and longitudinal HPV16 persistence.

BKPyV seropositivity	Yes	p-value
HPV variable	OR (95% CI)	
**Seropositivity to low risk HPV**	0.872 (0.324-2.352)	0.809
**Seropositivity to high risk HPV**	1.557 (0.535-4.531)	0.457
**Persistent genital HPV16 infection**	0.835 (0.285-2.443)	0.778
**Persistent oral HPV16 infection**	0.316 (0.061-1.633)	0.272
*JCPyV seropositivity*	*Yes*	
HPV variable	OR (95% CI)	
**Seropositivity to low risk HPV**	1.264 (0.811-1.970)	0.311
**Seropositivity to high risk HPV**	1.177 (0.747-1.853)	0.492
**Persistent genital HPV16 infection**	0.989 (0.599-1.634)	1.000
**Persistent oral HPV16 infection**	1.347 (0.656-2.764)	0.467

## 4 Discussion

Papillomaviruses were originally grouped in the family Papovaviridae, but since 2002, papillomaviruses were designated as a distinct family, Papillomaviridae, in the 7th Report of the ICTV (van Regenmortel et al., 2000). Because the genome organization and viral structure of HPyV and HPV are very similar, it could be anticipated to discover some clinical associations between these polyomaviruses and HPV, because both viruses can infect head and neck region and genital tract.

Simultaneous presence of both BKPyV or JCPyV and HPV has been analyzed in genital, colorectal, head and neck, lung, and prostate lesions (Martini et al., 2004; Balis et al., 2007; Giuliani et al., 2007; Giuliani et al., 2008; Comar, 2011; Fraase et al., 2012; Alosaimi et al., 2014; Polz-Gruszka et al., 2015; Poluschkin et al., 2018). Accordingly, BKPyV has been detected in HPV-positive and -negative cervical swabs and in precancerous lesions of the cervix (Comar, 2011; Fraase et al., 2012). BKPyV has also been found in HPV16-positive high-grade squamous intraepithelial neoplasia (CIN) lesions but not in low-grade CIN or normal cervical epithelium. Furthermore, BKPyV DNA copy number was higher in high-grade CIN suggesting that the presence of HPV could increase the BKPyV replication (Comar, 2011). In the present study, we analyzed the association between BKPyV and HPV using also high stringency cut-off (increase from 400 MFI to 5000 MFI) for BKPyV seropositivity to identify the women with possible BKPyV replication. Not even by this, we could not find any association between cervical cytology or CIN and BKPyV seropositivity. JCPyV has been detected both in low and high-grade CINs, but these infections could not be associated with HR-HPVs (Comar, 2011; Alosaimi et al., 2014).

Contrary to our findings in the uterine cervix, JCPyV might be more common in head and neck carcinomas than BKPyV (Poluschkin et al., 2018). JCPyV DNA was found in 86% of the HPV-positive tumors as compared to 14% of the HPV-negative lesions (Poluschkin et al., 2018). The same group identified only one BKPyV positive oral carcinoma (1/29), while none of the oropharyngeal carcinomas tested BKPyV DNA- positive by PCR (Poluschkin et al., 2018). Contradictory to that, Polz-Gruszka and coworkers reported HPV and BKPyV co-infection in 5% of 62 oropharyngeal carcinomas (Polz-Gruszka et al., 2015).

In the absence of longitudinal studies on this topic, our results are of particular interest. In this setting among healthy women, it was shown that being BKPyV and/or JCPyV seropositive was not associated with any of the HPV-related variables, including HPV-seropositivity to LR- or HR-HPVs, LR- or HR-HPV DNA detection, HPV16 persistence in the oral or genital mucosa, Pap smear abnormalities or incident CIN in the biopsy samples. When stratified by these HPV-related covariates, in practically all categories, over 90% of the women were BKPyV seropositive while seropositivity to JCPyV ranged from 52% to 68%, with no significant differences between strata.

It is known that HPyVs, especially BKPyV and JCPyV are ubiquitous in populations. In healthy adult population, BKPyV seroprevalence is around 90% while that of JCPyV is lower, around 50-70% (Hirsch and Steiger, 2003; Kean et al., 2009; Antonsson et al., 2010). In our previous study, seroprevalence in the present cohort of pregnant women was 94% for BKPyV and between 59%-68% for JCPyV (Laine et al., 2023). Also the mean IgG serum levels of BKPyV and JCPyV were high, but nearly 4-fold higher for BKPyV than for and JCPyV. Thus, the higher BKPyV seroprevalence (>90%) and lower seroprevalence of JCPyV (52% to 68%) in the present cohort is in line with the published data on healthy adult populations. At baseline, 53%, 21%, 35%, 21%, and 9% of these women were seropositive for HPV 6, 11, 16, 18, and 45, respectively (Syrjänen et al., 2009). Given that seropositivity to LR-HPV was more prevalent, one would expect some differences in HPyV seroprevalence when the cohort was stratified by LR-HPV and HR-HPV. However, this was not the case while no differences between LR-HPV+ and HR-HPV+ were found in their associations with HPyV seroprevalence. This precludes any definite conclusions on the mutual interactions between these two viruses (HPV and HPyV).

Some previous studies have focused on etiological association between HPyVs and HPV in cancer patients. However, the results are mostly in line with those observed in the present study, showing no associations between these two virus groups even in cancer patients. Martinez-Fierro and coworkers analyzed the presence of DNA of BKPyV, JCPyV, and HPV in 130 subjects: 55 prostate cancer patients and 75 controls. HPV sequences were detected in 11 (20%) of the cases and 4 (5%) controls. BKPyV, JCPyV, and SV40 sequences were not detected in any of the samples examined (Martinez-Fierro et al., 2010). Llewellyn and coworkers analyzed 689 fresh-frozen urothelial bladder carcinoma samples to detect HPV16 and HPV18 in addition to BKPyV/JCPyV using qPCR. Only one sample was positive for HPV16, while polyomaviruses were detected in 49 samples (Llewellyn et al., 2018). Giuliani and coworkers analyzed fresh tumor samples from 78 lung cancer patients for the presence of SV40, BKPyV, JCPyV, HCMV, and HPV sequences using PCR (Giuliani et al., 2007). Of these samples 10 were HPV-positive, while BKPyV and JCPyV were found only in one sample each.

In the present study, 90% and 59% of the women with persistent genital HPV16 were BKPyV and JCPyV seropositive, respectively. However, the distribution of BKPyV and JCPyV seroprevalence was similar in HPV16-infected women, who had cleared their HPV infection. In addition, no association was found between Pap smear abnormalities or incident CIN and BKPyV and/or JCPyV serostatus. Interestingly, Castellsague et al. showed that seropositivity to cutaneous HPV L1 or seropositivity to four polyomaviruses (including JCPyV) did not associate with CIN/CIS or cervical cancer (Castellsagué et al., 2014). In contrast, BKPyV has been detected in HPV-positive and -negative cervical swabs as well as in precancerous lesions of the cervix (Comar, 2011; Fraase et al., 2012). Similarly, Comar et al. showed BKPyV in CIN lesions together with HPV16 but not in low-grade CIN or normal cervical epithelium. Furthermore, JCPyV has been detected in high-grade and low-grade CINs but no association with HR-HPV infection was found (Comar, 2011; Alosaimi et al., 2014). Phenomenon termed superinfection exclusion explains why especially closely related virus genome could enter the cell infected by another virus but cannot establish a new infection (Hampson et al., 2020). Superinfection exclusion is studied among different HPV genotypes but not HPyVs (Hampson et al., 2020). Earlier studies with HPV positive cell cultures indicate that it is nearly impossible to re-infect these cells with another HPV genotype. We have shown *in vivo* that the first HPV16 to come established the persistent infection but the next HPV16 with slight genomic changes was able to cause only a transient HPV infection (Kurvinen et al., 2000). Thus, based on these earlier studies superinfection of HPV infected cells with HPyVs might not be possible since it seems that not even different HPVs could infect the same cells. Noteworthy is that in our study, none of the women were vaccinated to HPV which makes this study population very unique without any confounding factors.

One limitation of our study is that HPyV DNA analysis was not performed in the samples used for HPV-genotyping (oral and genital brush samples and CIN lesions). In addition, the number of these samples is low. In the clinical diagnostics, e.g. HPV serology has limited value and HPV seropositivity is usually regarded as a sign of past history of HPV infection. However, serological analysis is the only current method to study the possible association between HPV and HPyV types. *In vitro* models are not available as there are neither BKPyV nor JCPyV positive epithelial cell lines and HPV can not be cultured. The ultimate strength of the study is that the study design is novel and innovative which in the best case scenario would encourage other research groups to study the current issue within their own cohorts and especially utilizing *in situ* hybridization methods to localize these viruses in same cells progressing toward malignancy.

In conclusion, BKPyV and/or JCPyV seropositivity had no significant associations with any of the HPV-related covariates included in the present analysis. Thus, the present study could not provide any additional support to the concept that co-infections by HPyV and HPV have interactions that affect the clinical manifestations or outcomes of HPV infections either in the genital tract or in the oral mucosa.

## Data availability statement

The original contributions presented in the study are included in the article/supplementary material. Further inquiries can be directed to the corresponding author.

## Ethics statement

The studies involving human participants were reviewed and approved by Research Ethics Committee of Turku University Hospital. The patients/participants provided their written informed consent to participate in this study.

## Author contributions

HL, TW, KS, SG, KL, and SS contributed to the study conception and SS created the study design. Material preparation and data collection were performed by SS and TW. The analysis was performed by HL, SS, and KS. The first draft of the manuscript was written by HL and SS. All authors commented on previous versions of the manuscript and read and approved the final manuscript. All authors contributed to the article and approved the submitted version.
